# Levels of health literacy and English comprehension in patients presenting to South African primary healthcare facilities

**DOI:** 10.4102/phcfm.v12i1.2047

**Published:** 2020-01-30

**Authors:** Zelda Janse van Rensburg

**Affiliations:** 1 Department of Nursing, University of Johannesburg, Johannesburg, South Africa

**Keywords:** REALM-R, health literacy, primary healthcare facilities, Literacy, English comprehension, Learning Ability Battery (LAB)

## Abstract

**Background:**

Health literacy is a relatively new concept in the South African primary healthcare (PHC) sector as well as globally, and limited new literature is available on the topic. In this study, we focused on investigating, describing and comparing health literacy scores calculated using three different tools to assess a patient’s level of English comprehension. Health literacy is defined as the degree to which patients have the capacity to obtain, process and understand basic health information and services to make appropriate health decisions. South Africa is a linguistically and culturally diverse country, yet English is often used as the main language for imparting health education in PHC facilities. Patients often do not comprehend the health education received. Primary healthcare workers need to determine the health literacy levels of their patients before imparting health education. The REALM-R (SA 1, 2 and 3) tools are adapted from the original REALM-R to test health literacy levels of South African PHC patients. The Learning Ability Battery (LAB) is a tool used to determine English comprehension levels.

**Aim:**

The aim of this was to investigate, describe and compare health literacy and English comprehension levels of PHC patients using three locally adapted REALM-R (SA) tools and the LAB.

**Setting:**

This study was conducted at five PHC facilities in the City of Tshwane, Gauteng.

**Methods:**

A prospective, quantitative and comparative design was chosen for this study. In general, a descriptive design was applied for the presentation of the results. The sample size was 200 patients from five different PHC clinics. Data were collected using REALM-R (SA 1, 2 and 3) to determine the health literacy levels and the LAB to determine the English comprehension levels.

**Results:**

Majority of the patients scored high using the REALM-R (SA) tools. For the LAB, 68% scored 11–40 out of 50. Only 8% scored 41–50 out of 50. A significant difference was found between the actual school grade achieved and the school grade according to the LAB.

**Conclusion:**

The results of the study indicated that although patients are able to read and pronounce medical words as such used in the REALM-R (SA) tools, it does not necessarily mean that they are able to comprehend the meaning of the words as indicated by the results of the LAB. Currently, the REALM-R (SA) tools only test health literacy levels based on word recognition and pronunciation. It is recommended that a word comprehension section be added to determine patients’ understanding of the words.

## Introduction and background

Health literacy is a relatively new concept in the South African primary healthcare (PHC) sector as well as globally, and limited new literature is available on the topic. In this study, we focused on investigating, describing and comparing health literacy scores calculated using three different tools to assess a patient’s level of English comprehension. This article is part of a larger study forming part of a doctoral thesis.

Health literacy is defined as the degree to which patients have the capacity to obtain, process and understand basic health information and services to make appropriate health decisions.^1^ The National Adult Literacy Survey (NALS) found that a person’s literacy levels influence one’s ability to access information and function in the modern world.^1^ Low literacy levels refer to the inability to read, write and speak English, and compute and solve problems at levels of proficiency necessary to function in a working environment as well as in society to achieve one’s goals and develop one’s knowledge and potential.^2^ A general household survey conducted in South Africa in 2015 found that^3^ 7.1% of South Africans were unable to read, write or use numbers effectively. These low literacy levels may be linked to several adverse health outcomes.^4^ These include non-adherence to important instructions for taking prescribed medication,^5^ non-compliance with instructions for prevention and management of chronic diseases such as hypertension and diabetes,^6^ increased morbidity and mortality rates,^7^ increased hospitalisation and longer hospital stays^6^ and negative health behaviours such as smoking, abuse of illegal substances and other poor lifestyle choices.^8^

Taking the above facts into account, in a developing country such as South Africa, health education in either oral or written form is important especially in PHC facilities where the emphasis is on the promotion of health and prevention of diseases.^2^ South Africa is a linguistically and culturally diverse country, yet English remains the dominant language of communication in the public sphere. In PHC facilities in South Africa, English is often used as the main language for communicating with patients as well as for imparting health education either orally or in the form of handouts, brochures and posters or both.^1,2^ Such education plays an essential part in the promotion of health and prevention of diseases.^2^ This said, many patients may lack the ability to comprehend what they hear or read because the average person’s actual English comprehension level is five grades below that of the highest level of schooling achieved.^2^ There is often a mismatch between the patient’s highest level of schooling obtained and their literacy level.^1^ Health education materials are often written at a level higher than the patients understanding and the actual school grade achieved does not accurately reflect a patient’s ability to understand written health education materials given to them.^1^

Although the ability to read and comprehend health education materials is of high importance, patients’ health literacy and English comprehension levels are often overlooked when health education materials are designed and developed.^9^ Healthcare workers (HCWs) are also often unaware of the health literacy and English comprehension levels of their patients and some patients may be ashamed to mention that they do not understand the health information given.^10^ In order for PHC workers to give valuable health education orally or in written form, they first need to determine the health literacy and English comprehension levels of their patients.

Although HCWs assume that the health education and instructions given to the patient are readily understood, the reality is that these instructions are often misunderstood and can sometimes lead to serious medical errors.^1^ When health education is delivered, it should be tailored to the patient’s needs,^11^ such as imparting education in the patient’s mother tongue may give a more clear understanding of the intended message.^11^ If patients receive health education at their level of understanding, they are more likely to manage their medical conditions more effectively and to participate in decision-making about their health, adhere to prescribed medication and live a healthier lifestyle, all of which are likely to lead to improved health outcomes.^5^

## Research design and method

### Study design

A prospective, quantitative and comparative design was chosen for our study. A prospective design refers to the researcher starting with a presumed cause and then going forward in time to the presumed effect.^12^ The prospective design was necessary as there was no existing data set available for analysis that adequately spoke to the aim and objectives of the study.^12^ Quantitative research involves the investigation of a phenomena, getting precise measurements and quantifying the results.^12^ The study was quantitative as the data sets emerging from the tools (REALM-R SA 1, 2 and 3) and the Learning Ability Battery (LAB) were quantitative in nature.^12^ A descriptive design entails using statistics to describe and summarise the data.^12^ In general, a descriptive design was applied for the presentation of the results.

### Setting

Data were gathered onsite at five PHC facilities in the City of Tshwane, Gauteng. These clinics were purposively selected by the researcher from the Tshwane metropolitan municipality because of their accessibility to the researcher. Vulnerable patients such as those who were mentally and emotionally disabled and severely ill were not approached to participate in this study.^12^ The clinics included two from North Tshwane, one from central Tshwane, one from West Tshwane and one from South Tshwane. The average number of patients per clinic is 73 117 patients per month.

### Population and sampling strategy

Following statistical consultation, a convenience sample of 200 patients (40 from each of the five clinics) was determined as sufficient to meet the aims and objectives of the study. The researcher advisor template was used by the statistician. The REALM-R (SA) was further validated as part of the larger study on 400 patients; however, it was not included in this article. The inclusion criteria were as follows: participants must be South African citizens, must be between 18 and 60 years old and have a self-declared ability to be able to read English. Prospective participants were approached by the researcher on the day they attended the clinic and those who were willing to participate in the study and met the inclusion criteria were included in the study.

### Tools and data collection

Three versions of the REALM-R (SA)^9^ and the LAB^13^ were used to assess the patients’ English language and literacy levels. In this section, each of these tools is described in turn.

### The REALM-R (SA)

**FIGURE 1:**
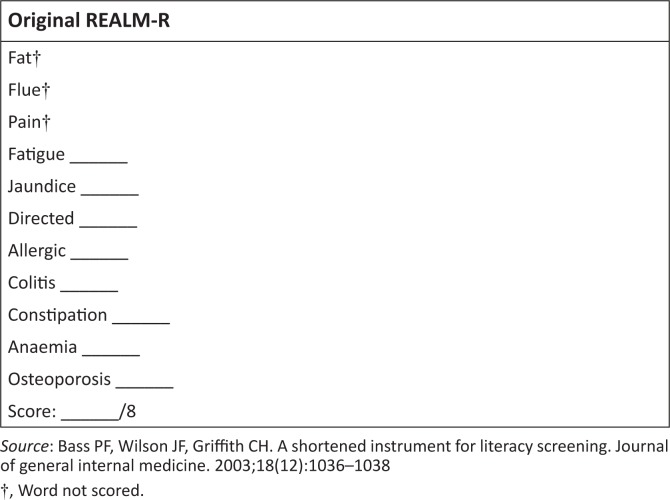
The original REALM-R tool.

The REALM-R was originally developed in the United States as a health literacy assessment tool based on word recognition and pronunciation.^9^ The eight words of the original REALM-R are indicated in Figure 1.^9,12^

The original REALM-R was locally adapted in a pilot study^14^ to three different REALM-R (SA) tools by using a modified Delphi Technique. The original REALM-R was also similarly adapted in a previous study by Mafutha, Mogotlane and de Swardt^15^ to assess hypertension-related literacy levels of PHC patients. In the pilot study, the original words of the REALM-R were changed to words more frequently used in the South African PHC facilities.^14^ In the pilot study, only one of the REALM-R (SA) tools was implemented.^14^

In this study, all three locally adapted REALM-R (SA) tools were used to assess the health literacy levels of South African PHC patients in the five selected PHC facilities. Similar to the original REALM-R, the three REALM-R (SA) tools required the participant to read and pronounce a series of 11 English words at different levels of complexity.^16^ The first three words such as *pain, sick* and *food* are relatively easy words, and were not scored but used to set the participant at ease before continuing with the other words. Only the last eight words were scored and each word that was pronounced correctly was attributed a mark.^16,17^ The second set of words such *condom, fever, infection, transmission* and *prevention* were words that are commonly used in the PHC facilities and were more difficult to pronounce. The last three words such as *contraception, immunisation* and *immunocompromised* were even more difficult to pronounce. A final score out of eight was allocated at the end of the assessment. Similar to the original REALM-R, patients with a score of 6 or lower out of 8 were considered to have low medical literacy levels.^18^ The data were collected by three fieldworkers who received 2 days of training before implementing the tools.

Each of the 200 participants was assessed using all three REALM-R (SA) tools (REALM-R SA 1, 2 and 3). The assessment was conducted in a private room at the clinic to prevent psychological discomfort when the participant was not able to pronounce a word. The scoring was done sequentially. The accuracy of the English word recognition and pronunciation was noted by the fieldworker and scored accordingly. For the scoring, the fieldworkers indicated a plus sign (+) after each word that was pronounced correctly. A zero (0) was indicated after words that were pronounced incorrectly. A minus sign (-) after words showed that there was no attempt made at pronunciation. Self-corrected words were marked with a plus sign (+) if correct and were counted as such. If the participant hesitated for more than 5 s, the fieldworker would say ‘pass’ and ask the participant to continue to the next word. This missed word would be marked with a ‘0’. The participant received one opportunity to self-correct mispronounced words before the total score was calculated. The plusses were counted and a score out of 8 was indicated for each participant on a raw score sheet. The data gathering took approximately 2–3 min per REALM-R (SA). Table 1^14^ shows the three REALM-R (SA) tools and the words used in each assessment.

**TABLE 1:** The three versions of REALM-R (SA) tools.

REALM-R (SA 1)	REALM-R (SA 2)	REALM-R (SA 3)
Pain†	Food†	Germs†
Sick†	Germs†	Pain†
Food†	Pain†	Food†
Condom ______	Treatment ______	Condom _____
Fever ______	Condom ______	Fever _____
Infection ______	Transmission ______	Infection _____
Transmission ______	Vomiting ______	Prevention _____
Prevention ______	Prevention ______	Transmission _____
Contraception ______	Hypertension ______	Constipation _____
Immunisation ______	Tuberculosis ______	Antibiotics _____
Immunocompromised______	Osteoporosis ______	Gastroenteritis _____
Score: ______/8	Score: _____/8	Score: ______/8

*Source:* Wassermann Z, Wright SCD, Maja TM. Adaptation of the Rapid Estimate of Adult Literacy in Medicine Revised (REALM-R) to the South African context: Part 1. Health SA Gesondheid. 2010;15(1):77–81

† , Word not scored.

### The Learning Ability Battery

To assist in determining the English comprehension levels of South African PHC patients, they were also assessed using the LAB. The LAB is a tool that identifies a person’s English comprehension levels based on the South African school grade levels (grades 1–12).^13^ The LAB assessment included an English language section based on word recognition and comprehension.^16^ The patients were required to read and complete sentences, ranging in levels of difficulty and complexity (sections 1–5) according to school grade levels. Participants needed to choose the most correct word from the words enclosed in brackets at the end of each sentence to complete the sentence (Box 1).^18^

BOX 1 Correct word from the words enclosed in brackets at the end of each sentence to complete the sentence.

Section 1: When you cook you use this (*hot, hat, pot, pat*).
Section 2: This shirt is one size too (*lark, leg, last, large*).
Section 3: The children will (*sink, sing, sung, song*) *a new song in the concert.*
Section 4: Mr Smith is limping because he slipped and twisted his *(amlet, ankle, neck, umbrella*).
Section 5: Wild animals living close to people must have some (*charisma, charming, characteristic, enchantment*).

*Source:* Lead the Field. Learning Ability Battery (LAB). Randburg: Lead the Field Training Office; 2012

Each section consisted of 10 English sentences. The participants were allowed to complete the LAB in a private room to decrease disturbances. The LAB took approximately 30–40 min to complete per patient. A total score out of 50 was allocated and entered into a computer program, called WinLAB, which produced each participant’s word recognition and language ability according to the National Qualifications Framework (NQF) levels.^13^ A comparison between the results of the three different REALM-R (SA) tools and the LAB was done after completion. Each participant was also asked to enter their highest level of schooling achieved on the LAB before completion. A comparison of the actual school grade level according to the LAB results and the highest level of schooling as indicated by the participant was compared later (Table 3).

### Data analysis

The completed assessments were analysed by a statistician using STATA V12 statistical software. The analysis enabled comparison of the results of the three versions of the REALM-R (SA) tools which are presented in Table 2 in the ‘Results’ section. A comparison between the highest level of schooling completed and the actual school grade level according to the LAB was also done (Table 4).

**TABLE 2:** Comparison of the results of the three REALM-R (SA) tools.

REALM-R score	Number of respondents					
	REALM-R (SA 1)		REALM-R (SA 2)		REALM-R (SA 3)	
	*n*	%	*n*	%	*n*	%
0	0	0.0	0	0.0	2	1.0
1	1	0.5	1	0.5	2	1.0
2	2	1.0	2	1.0	1	0.5
3	2	1.0	1	0.5	0	0.0
4	3	1.5	3	1.5	2	1.0
5	16	8.0	12	6.0	13	6.5
6	29	14.5	27	13.5	30	15.0
7	46	23.0	71	35.5	118	59.0
8	101	50.5	83	41.5	32	16.0
**Total**	**200**	**100.0**	**200**	**100.0**	**200**	**100.0**

### Ethical considerations

Permission to use the tools with patients was granted by the Research Ethics Committee of the University (REF# REC2012/11/009(2)) as well as by the Tshwane Metropolitan Municipality. The respondents were informed about the purpose of the study and how it was going to be conducted and signed an informed consent document prior to the assessments. The respondents had the right to withdraw from the study at any time without any negative impact on them. A number was allocated to each respondent and the name of the respondent was kept confidential. The age and highest level of schooling achieved were also not disclosed to anyone except the researcher and the fieldworkers. The assessments of the REALM-R (SA) tools were conducted in a private room at the relevant PHC facility to maintain privacy and prevent psychological discomfort when the respondents were not able to pronounce a word.

## Results

### Demographics

The age of the participants ranged between 18 and 60 years. Of the 200 participants, 3/200 (1.5%) were between ages 18 and 19 years, 72/200 (36%) between ages 20 and 29 years, 65/200 (32.5%) between ages 30 and 39 years, 34/200 (17%) between ages 40 and 49 years and 20/200 (10%) between ages 50 and 59 years. Only 6/200 patients (3%) were 60 years old.

Table 2 indicates the results of patients’ scores using the three versions of the REALM-R (SA 1, 2 and 3) tools (Table 2) and the LAB (Table 3). A comparison of the participant’s highest level of schooling completed and the actual school grade level according to the LAB is presented in Table 4.

**TABLE 3:** Results of the Learning Ability Battery (*n* = 200).

LAB score	Number of respondents		Combined scores	
	*n*	%	*n*	%
< 10	16	8	16	8
11–20	32	16	168	84
21–30	76	38	-	-
31–40	60	30	-	-
41–50	16	8	16	8
**Total**	**200**	**100**	**200**	**100**

As shown in Table 2, for the REALM-R (SA 1), 50.5% scored 8 out of 8 and 26.5% (53) scored 6 and below. For REALM-R (SA 2), 41.5% (83) scored 8 out of 8, while 23% (46) scored 6 and below. For the REALM-R (SA 3), 16% scored 8 out of 8, while a total of 25% (50) scored 6 and below.

Table 3 indicates that most of the participants (68%) achieved a score of 21–40 out of 50 for the LAB, while very few participants (8%) achieved a score of 41–50.

The participants were asked to indicate their highest level of schooling completed on the LAB assessment tool (see Table 4, column 2). After completion of the assessment using LAB, the achieved school grade level according to the LAB was entered into a computer-based programme. In Table 4, the highest level of schooling completed by the participants (column 2) and the actual school grade as achieved by the LAB (column 3) are compared. The results were compared to determine the difference of the school grade that the participants indicated they have completed and what their actual school grade was.

**TABLE 4:** Participant’s highest level of schooling completed compared to the school grade achieved by the Learning Ability Battery.

South African school grade levels	Participant’s highest level of schooling completed		School grade level achieved by the LAB	
	*N*	%	*N*	%
1–3	1	0.5	16	8.0
4–6	5	2.5	37	18.5
7–8	15	7.5	63	31.5
9–12	179	89.5	84	42.0
**Total**	**200**	**100.0**	**200**	**100.0**

LAB, Learning Ability Battery.

Table 4 indicates that although 89.5% of the participants indicated a highest level of schooling of grades 9–12 only 42% scored at this level as achieved by the LAB. The difference between the highest level of schooling completed and the actual school grade as achieved by the LAB was 3.35 grades.

## Discussion

In our study, we focused on investigating, describing and comparing patients’ health literacy scores using three different tools (REALM-R SA 1, 2 and 3) to assess their level of English comprehension (LAB). The greater number of participants were between the age of 20 and 29 years. Taking this into consideration, these participants would have formed part of the new South African schooling system and would have been expected to have achieved a grade 12 level of schooling. The analysis of the data indicates that while the majority of participants received high scores based on the assessment using the REALM-R (SA) tools (Table 2), their scores based on the LAB tool (Table 3), which assessed the respondents’ English reading and comprehension which indicates the actual school grade level of the participant, were somewhat lower. The LAB was used to assess the patient’s meaningful understanding of the English words at different school grade levels. Although the majority (89.5%) of the respondents declared a grade 11–12 level of schooling (Table 3), according to the LAB only 42% of the respondents scored at this level. A school grade difference of 3.35 was found between the patient’s highest level of schooling achieved and the actual school grade level in this study. Badarudeen and Sabharwal’s^9^ study supports this as they found that patients’ comprehension levels are usually three to five grades lower than their word recognition levels when confronted with health information. In a previous study conducted in South Africa,^14^ a school grade difference of four grades was found to exist. A school grade difference of 3.35 suggests that South African PHC patients should first be assessed on their health literacy levels and English comprehension levels before health education is imparted. Although PHC patients may be able to read and pronounce commonly used medical terms, they might not be able to comprehend the meaning of the words. Patients with low health literacy levels, as indicated by the scores of 6 or below out of 8 for the REALM-R (SA) tools, may find it difficult to comprehend most health education information either written or oral.^9,13^

Several authors have argued that a reading level below a grade 8 school level may lead to patients finding it difficult to comprehend most health education information (either written or oral).^9,13^ In this study, majority of the participants achieved a score of 7–8 out of 8 for the REALM-R (SA), with scores of 73.5% for REALM-R (SA 1), 77% for REALM-R (SA 2) and 75% for REALM-R (SA 3) (Table 1) and thus have high health literacy levels and are possibly able to understand health education information received in English. However, the rest of the participants received a score of 6 and below which is an indicator of low health literacy levels. A score at this level is equivalent to an English reading level of the eighth grade level and below.^9^ Most health education materials are prepared for an eighth grade level of schooling or higher and these patients may therefore not optimally benefit from health education materials currently available in the South African PHC facilities.^1^ At the PHC level, the communication between the HCW and the patient can be improved by the early determination of the patient’s health literacy levels.^1^ Although a score of 7–8 out of 8 indicates a school grade level of the eighth grade and above, the ability to comprehend health education information received in English can still be questioned.

Patients may often feel too embarrassed to indicate that they have not really understood instructions given by HCW or be too ashamed to indicate a low level of schooling achieved.^10^ The ability to read and comprehend either written or oral health education information is an important element in the prevention of disease and the promotion of health.^9^ These levels also need to be recognised when health education information is being prepared.^1^ Health education should not be written at a level higher than the sixth to eighth grade level^9^; however, at the PHC level many health education programmes do not meet these standards.^9^ At this level of healthcare, it is of great importance that health education material be designed to be in line with the health literacy levels of the patients^1^ and a tool such as the REALM-R (SA) can assist in determining this.

Patients with low levels of health literacy may find it difficult to understand health education information and health instructions and may be less compliant to treatment.^1^ An adequate level of health literacy means that the individual would be able to take responsibility for his or her own health as well as that of their family and adapt to healthy practices and reduce risk behaviours.^1^

The REALM-R (SA) only assesses health literacy levels of the patient based on their ability to recognise and pronounce medical words frequently used in the South African PHC facilities and does not test comprehension of the English words.^9^ The LAB was therefore used to determine the English comprehension levels of the patients. The REALM-R (SA) tools, however, only assess the health literacy levels of the patient based on medical terms often used in the South African PHC facilities and not the comprehension of the English word in general. The ability to read and comprehend written health education materials can affect a person’s ability to comprehend.^9^ The ability to comprehend health information given in English should therefore be further investigated and allows opportunity for future research. Furthermore, low health literacy levels may not only be detected through English comprehension levels but also as poor oral communication skills,^9^ for which a tool such as the REALM-R (SA) may not be sufficient. The REALM-R (SA) has only been adapted from the original tool and is only available in English. Because of the linguistic and cultural diversity of South Africa, it is recommended that the REALM-R (SA) be translated into other South African languages such as Sepedi, Setswana, Zulu, Xhosa and even Afrikaans. Furthermore, the REALM-R (SA) tools need to be further refined to correctly reflect the South African context to give a better reflection of the English reading and comprehension levels. Although the REALM-R (SA) was further validated on 400 patients, a future study with a larger sample is recommended. The LAB tool assesses English reading and comprehension levels, and is available in all 11 official languages, but it is a lengthy tool and takes approximately 30–40 min to complete and is therefore not ideal for use in a busy PHC facility. The applicability of other health literacy and English comprehension tools should also be investigated as only the REALM-R (SA) and the LAB were used in this study. Low English reading and comprehension levels are associated with poor health outcomes. This association allows opportunity for future research such as patient’s health literacy levels and English comprehension related to the adherence to prescribed medicine. It is also recommended that better health education should be established through the implementation of health literacy guidelines and policies at the national level.^19^ The incorporation of health literacy in the curricula of health sciences students is also recommended.

## Conclusion

Although the participants received good scores using the REALM-R (SA) tools, the results of the LAB test indicated poorer results. It is evident that although the participants can read and pronounce the medical words of the REALM-R (SA) tools, they lack comprehension of the meaning of the English word. A significant difference between the highest level of schooling completed and the actual school grade level according to the results of the LAB was found. The implication of this is that although patients may indicate a high level of schooling completed, they may not actually understand health education imparted at that level. A determination of the patient’s health literacy levels and English comprehension levels is suggested before health education is given to the patient in order for health education to be given on the patient’s optimal level of understanding leading to patient-centred communication. Although tools, such as the REALM-R (SA), can be used to determine the medical literacy levels of PHC patients, it needs further refinement and should be further validated on a bigger population.
